# Urinary miR-29 Correlates with Albuminuria and Carotid Intima-Media Thickness in Type 2 Diabetes Patients

**DOI:** 10.1371/journal.pone.0082607

**Published:** 2013-12-09

**Authors:** Hui Peng, Meirong Zhong, Wenbo Zhao, Cheng Wang, Jun Zhang, Xun Liu, Yuanqing Li, Sujay Dutta Paudel, Qianqian Wang, Tanqi Lou

**Affiliations:** Department of Internal Medicine, Division of Nephrology, The Third Affiliated Hospital of Sun Yat-sen University, Guangzhou, Guangdong, P. R. China; University of Sao Paulo Medical School, Brazil

## Abstract

**Background:**

Cell-free microRNAs stably and abundantly exist in body fluids and emerging evidence suggests cell-free microRNAs as novel and non-invasive disease biomarker. Deregulation of miR-29 is involved in the pathogenesis of diabetic nephropathy and insulin resistance thus may be implicated in diabetic vascular complication. Therefore, we investigated the possibility of urinary miR-29 as biomarker for diabetic nephropathy and atherosclerosis in patients with type 2 diabetes.

**Methods:**

83 patients with type 2 diabetes were enrolled in this study, miR-29a, miR-29b and miR-29c levels in urine supernatant was determined by TaqMan qRT-PCR, and a synthetic cel-miR-39 was added to the urine as a spike-in control before miRNAs extraction. Urinary albumin excretion rate and urine albumin/creatinine ratio, funduscopy and carotid ultrasound were used for evaluation of diabetic vascular complication. The laboratory parameters indicating blood glucose level, renal function and serum lipids were also collected.

**Results:**

Patients with albuminuria (n = 42, age 60.62±12.00yrs) showed significantly higher comorbidity of diabetic retinopathy (p = 0.015) and higher levels of urinary miR-29a (p = 0.035) compared with those with normoalbuminuria (n = 41, age 58.54±14.40yrs). There was no significant difference in urinary miR-29b (p = 0.148) or miR-29c level (p = 0.321) between groups. Urinary albumin excretion rate significantly correlated with urinary miR-29a level (r = 0.286, p = 0.016), while urinary miR-29b significantly correlated with carotid intima-media thickness (cIMT) (r = 0.286, p = 0.046).

**Conclusion:**

Urinary miR-29a correlated with albuminuria while urinary miR-29b correlated with carotid intima-media thickness (cIMT) in patients with type 2 diabetes. Therefore, they may have the potential to serve as alternative biomarker for diabetic nephropathy and atherosclerosis in type 2 diabetes.

## Introduction

Microvascular and macrovascular complications are the major causes of mortality in people with type 1 and type 2 diabetes. Microvascular complications include nephropathy, retinopathy and neuropathy, while the macrovascular manifestations are consequences of accelerated atherosclerosis [1]. Furthermore, microvascular complications are associated with cardiovascular events in type 2 diabetes, and patients with albuminuria or reduced GFR had nearly two-fold increased risk for cardiovascular events. This phenomenon suggested that similar mechanisms may be involved in the pathogenesis of both micro- and macrovascular disease in type 2 diabetes [2]. The existence of common pathways such as oxidative stress and vascular remodeling may bring light to potential therapeutic targets as well as biomarkers for both diabetic vascular complications [3], [4] and foundation of new biomarkers for vascular and renal complications would provide clinically useful tools in diabetes care.

MicroRNAs (miRNAs) are a group of 19-25nt noncoding RNAs which regulate gene expression through post-transcriptional degradation of target mRNAs [5]. MiRNAs are involved in almost every aspects of cellular process and dysregulation of miRNAs was associated with many diseases including diabetic vascular complications [6]. Cell-free miRNAs abundantly exist in a variety of body fluids including serum, plasma, urine and saliva; their unique expression patterns are associated with specific physiological or disease condition [7]. Furthermore, cell-free miRNAs are shielded from degradation of RNase through packaging in exosomes, microvesicles and apoptotic bodies [8], or formation of protein-miR complexes with argonaute 2 (Ago2) or high-density lipoprotein (HDL)-associated protein [9], [10]. Therefore, cell-free miRNAs are revealed as a novel class of non-invasive disease biomarker with specificity, stability and reproducibility.

MiR-29 family is composed of miR-29a, miR-29b and miR-29c and they share the same seed sequence. Based on its potential to repress many kinds of collagens, the most extensively studied function of miR-29 was its protective role in fibrotic disease including kidney fibrosis [11]. MiR-29 is involved in pathogenesis of diabetic nephropathy by targeting Spry1 in db/db mice or collagen in STZ-induced diabetic mice [12], [13]. Furthermore, miR-29 is up regulated in muscle, fat and liver in type 2 diabetic rats and caused insulin resistance in adipocyte and the overexpression of miR-29 inhibits the activation of Akt which is a crucial mediator of insulin signal transduction [14]. Insulin signaling was considered a protective factor against diabetic vascular complication [15]. Podocyte-specific knockout of insulin receptor caused renal pathology changes similar to diabetic nephropathy and worsening of renal function in mice [16]. Complete loss of insulin signal in vascular endothelial cells aggravated atherosclerosis in endothelium-specific insulin receptor–deficient apolipoprotein E null mice [17]. Insulin resistance including endothelial insulin resistance was considered important determinant of atherosclerosis [18], [19]. These combined observations suggest that miR-29 might associate with vascular complications in type 2 diabetes. Moreover, urinary miRNAs can be derived from transrenal release or is excreted by every segment of nephron. Cheng at al reported that miRNAs containing exosomes can be excreted by kidney and heart-enrich miRNAs such as miR-1 and miR-208 were significantly increased in urine of animals and patients with acute myocardial infarction, thus suggesting urinary miR-1 as novel biomarkers for AMI [20].Therefore, urinary miRNAs may have the potential to serve as biomarkers of cardiovascular disease in addition to kidney disease. In this report, we explore the possibility of urinary miR-29 family as biomarker for diabetic nephropathy and atherosclerosis in patients with type 2 diabetes.

## Participants and Methods

### Participants

The study consists of 83 consecutive diagnosed type 2 diabetes patients admitted in endocrinology department after the exclusion of those with clinical and laboratory findings of urinary tract infection or malignant diseases. We recorded their previous history of cardiovascular events (a composite of angina pectoris, myocardial infarction, heart failure, occlusion of the retinal artery, arterial occlusion of lower extremities and stroke), comorbidity of hypertention (SBP/DBP>140/90 mmHg) and prescribed medications {glucose-lowering medication including metformin, sulfonylureas, thiazolidinedioness, alpha-glucosidase inhibitors, DPP-4 inhibitors and insulin; angiotensin-converting enzyme inhibitor(ACEI)/angiotensin II receptor blocker(ARB); statins and aspirin}. According to the level of albuminuria, they were divided into two groups: diabetes with albuminuria (Urinary albumin/creatinine ratio >30 mg/g, n = 42) and diabetes with normoalbuminuria (Urinary albumin/creatinine ratio <30 mg/g, n = 41). Mid-stream urine samples (nearly 10 mL) were collected in the morning from all the subjects. The study was approved by the Ethics Committee of the third affiliated hospital of Sun Yat-sen University (Guangzhou, China) and written informed consent was obtained from each subject.

### Sample Preparation

Urine specimen was collected and immediately centrifuged at 3000 g for 30 min at room temperature and then centrifuged at 13000 g for 5 min at 4°C. The urine supernatant of each subject was stored at −80°C until analysis for cell-free miRNAs expression.

### RNA isolation and Quantification of miRNA Level

The urine microRNA purification kit (Norgen, Thorold, Canada) was used for the isolation of miRNA in the urine supernatant according to the manufacturer’s protocol. 1 mL of urine supernatant was used for extraction of miRNA from each urine specimen. Prior to extraction step, 100 fmol/ml of synthesized non-human miRNA: cel-miR-39(Qiagen, Hilden, Germany) was added into equal volume of urine samples to serve as a spike-in control for normalization. Reverse transcription was performed with reverse transcription kit (Applied Biosystems, Foster City, CA). 3 uL specific stem-loop primers was added to the mixture of 1 uL (50 U) MultiScribe reverse transcriptase, 1.5 uL 10×reverse transcription buffer, 0.15 uL 100 mM dNTPs (with dTTP), 0.19 uL RNase inhibitor,5 uL RNA (20 U/uL) as well as 7.16 uL DEPC H_2_O to make a total volume of 15 uL. Reverse transcription was carried out at 16°C for 30 min, 42°C for 30 min and 85°C for 5 min. The synthesized complementary DNA was used for quantitative polymerase chain reaction immediately or stored at 4°C overnight.

Urinary expression of miR-29a, miR-29b, miR-29c were quantified by real-time quantitative polymerase using the same ABI 7500 Sequence Detection System. All primers used were obtained from Applied Biosystem Company (Carlsbad, CA) including cel-miR-39. For RT-QPCR, 1 uL primer and probe was mixed with 1.33 uL cDNA, 10 uL universal master mix, and 7.67 uL DEPC H_2_O to acquire a reaction volume of 20 uL. RT-QPCR was run at 50°C for 2 min, 95°C for 10 min, followed by 40 cycles at 95°C for 15 s and 60°C for 1 min. Each sample was performed in triplicate including no template controls. A comparative ΔCT method was used to compare each target with cel-miR-39, and relative values were expressed as 2^−ΔCT^. Data analysis was based on relative abundance of miR-29 family.

### BMI, Laboratory Parameters, Quantitative Sensory Testing (QST) and Funduscopy

BMI  =  Weight (kg)/Height (m)^2^, Serum levels of fructosamine and glycosylated hemoglobin (HbA1C) was tested on Olympus AU640 (Japan) and Bio-Rad D-10( CA, USA) respectively, serum creatinine, urea nitrogen, cystatin, β2-microglobulin,cholesterol, triglycerides (TGs), high-density lipoprotein-cholesterol (HDL-C), low-density lipoprotein-cholesterol (LDL-C),as well as the urinary albumin and creatinine content were detected on a Hitachi 7180 analyzer(Japan) with commercial reagents. The CKD-EPI (Chronic Kidney Disease Epidemiology Collaboration) creatinine equation was used for GFR estimation [21]. Funduscopy and quantitative sensory testing were performed in each of the enrolled patients during hospitalization in order to screen for diabetic retinopathy and diabetic peripheral neuropathy.

### Carotid Ultrasound Assessment

The extent of carotid atherosclerosis was evaluated by specialists in ultrasound department with high-resolution B mode ultrasonography. They first scanned the right and left common carotid artery and carotid sinus as high up as possible to examine the sclerotic lesions. The carotid intima-media thickness (cIMT) was measured as the distance from the leading edge of the lumen–intima interface to the leading edge of the collagenous upper layer of the adventitia on common carotid artery 10 mm proximal to the bifurcation. The average of three measurements (anterolateral, lateral, and posterolateral) of cIMT value was used for study.

### Statistical Analyses

The statistical analyses were performed with SPSS ver13.0 (SPSS, Chicago, IL, USA). Continuous variables are presented as means±standard deviation while non-parametric variables are expressed as median and interquartile range. Continuous variables were tested by the Student’s test or Mann-Whitney U test, chi-square test was used for comparison of categorical variables. The relationship between two continuous variables was assessed by a bivariate correlation method (Spearman’s rank order correlations). Statistical significance was defined as a p value <0.05. All probabilities were two-tailed.

## Results

The demographic data, laboratory parameters, comorbidities and complications, and prescribed medication were summarized in Table 1. There was no significant difference in age, gender, duration of diabetes, previous history of cardiovascular events, comorbidities of peripheral neuropathy, serum glycosylated hemoglobin levels, cIMT and prescription of glucose-lowering medication, ACEI/ARB, statins and aspirin between two groups. The diabetes with albuminuria group had higher prevalence of hypertention (p = 0.013), higher comorbidity of diabetic retinopathy (p = 0.015), higher triglycerides level (p = 0.007) and declined renal function, which meant higher levels of urea, creatinine, cystatin, β2-microglobulin(all with a p value<0.001) and lower eGFR (p<0.001) compared with the results of diabetes with normoalbuminuria group. Funduscopy of all the 83 participants showed no sign of hypertensive retinopathy.

**Table 1:** Characteristics of 83 participants.

Variables^a^	DM with normoalbuminuria (n = 41)	DM with Albuminuria (n = 42)	P
Sex(M/F), n	27/14	20/22	0.094
Age, years	58.54±14.40	60.62±12.00	0.208
Duration of DM, years	6.87±5.74	8.29±6.17	0.364
BMI	23.29±0.62	25±0.78	0.089
fructosamine, mg/dL	247.71±78.40	245.23±95.52	0.897
HbA1C, %	9.05±2.85	9.11±2.82	0.769
Urea, mg/dL	33.52±8.97	55.38±40.60	<0.001**
Creatinine, mg/dL	0.77±0.22	1.70±1.48	<0.001**
Cystatin, mg/dL	0.095±0.032	0.164±0.087	<0.001**
TC, mg/dL	155.07±40.60	187.94±49.88	0.657
TGs, mg/dL	138.18±71.74	149.78±98.32	0.007*
HDL-C, mg/dL	40.99±11.99	38.67±14.69	0.737
LDL-C, mg/dL	104.02±34.80	109.05±42.92	0.566
eGFR, mL/min/1.73 m^2^	95.77±20.47	62.58±38.26	<0.001**
UACR, mg/g	31.59±106.37	291.24±611.84	0.003*
UAER, ug/min	19.22±45.22	203.32±398.41	<0.001**
cIMT, mm	0.91±0.22	0.88±0.26	0.897
Comorbidity and complication			
Previous cardiovascular events, n (%)	6 (14.6)	13 (30.9)	0.077
Hypertention, n (%)	17 (41.5)	35 (83.3)	0.013*
Retinopathy, n (%)	6 (14.6)	16 (38.1)	0.015*
Peripheral neuropathy, n (%)	11 (26.8)	13 (30.9)	0.679
Prescribed medication			
Metformin, n (%)	23 (56.1)	16 (38.1)	0.100
Sulfonylureas, n (%)	23 (56.1)	15 (35.7)	0.062
Thiazolidinedioness, n (%)	2 (4.9)	2 (4.8)	0.683
Alpha-glucosidase inhibitors, n (%)	13 (31.7)	9 (21.4)	0.289
DPP-4 inhibitors, n (%)	2 (4.9)	2 (4.8)	0.683
Insulin, n (%)	27 (65.9)	33 (78.6)	0.196
ACEI/ARB, n (%)	13 (31.7)	32 (47.6)	0.139
Statins, n (%)	14 (34.1)	10 (23.8)	0.299
Aspirin, n (%)	17 (41.5)	11 (26.2)	0.141

DM = Diabetes mellitus, BMI = body mass index, HbA1C = glycosylated hemoglobin, TC = Total cholesterol, TGs = triglycerides, HDL-C = high-density lipoprotein-cholesterol, LDL-C = low-density lipoprotein-cholesterol, eGFR  = estimated glomerular filtration rate, UACR = urinary albumin creatinine ratio, UAER = urine albumin excretion rate, cIMT = carotid intima-media thickness, ACEI = angiotensin converting-enzyme inhibitor, ARB = angiotensin II receptor blocker.

aTo convert cystatin and β2-microglobulinconcentrations from mg/dL to mg/L, multiply by 10. To convert urea concentrations from mg/dL to mmol/L, multiply by 0.1665. To convert creatinine concentrations from mg/dL to mmol/L, multiply by 88.402. To convert total cholesterol, high-density lipoprotein-cholesterol, and low-density lipoprotein-cholesterol concentrations from mg/dL to mmol/L, multiply by 0.02586. To convert triacylglycerol concentrations from mg/dL to mmol/L, multiply by 0.01129. *p<0.05, **p<0.001.

### Urinary miR-29 Family Levels

**Figure 1:**
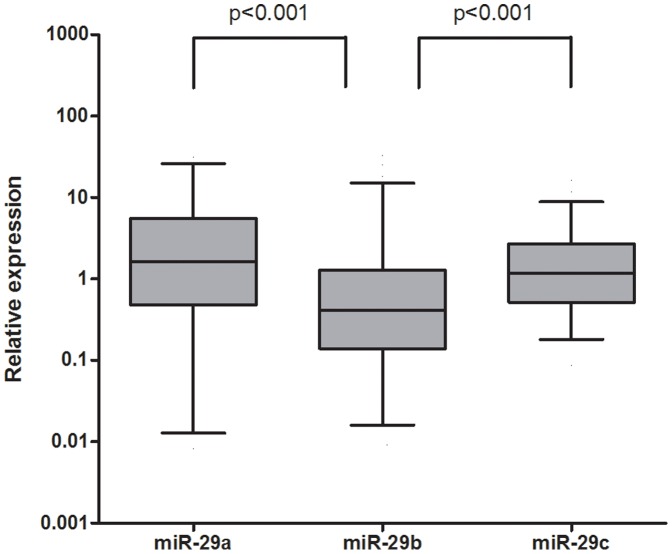
The relative abundance of urinary miR-29 members in patients with Type 2 diabetes mellitus (n = 83). Urinary miR-29a and miR-29c are significantly higher than miR-29b in type 2 diabetes patients (both with a p value <0.001).

**Figure 2:**
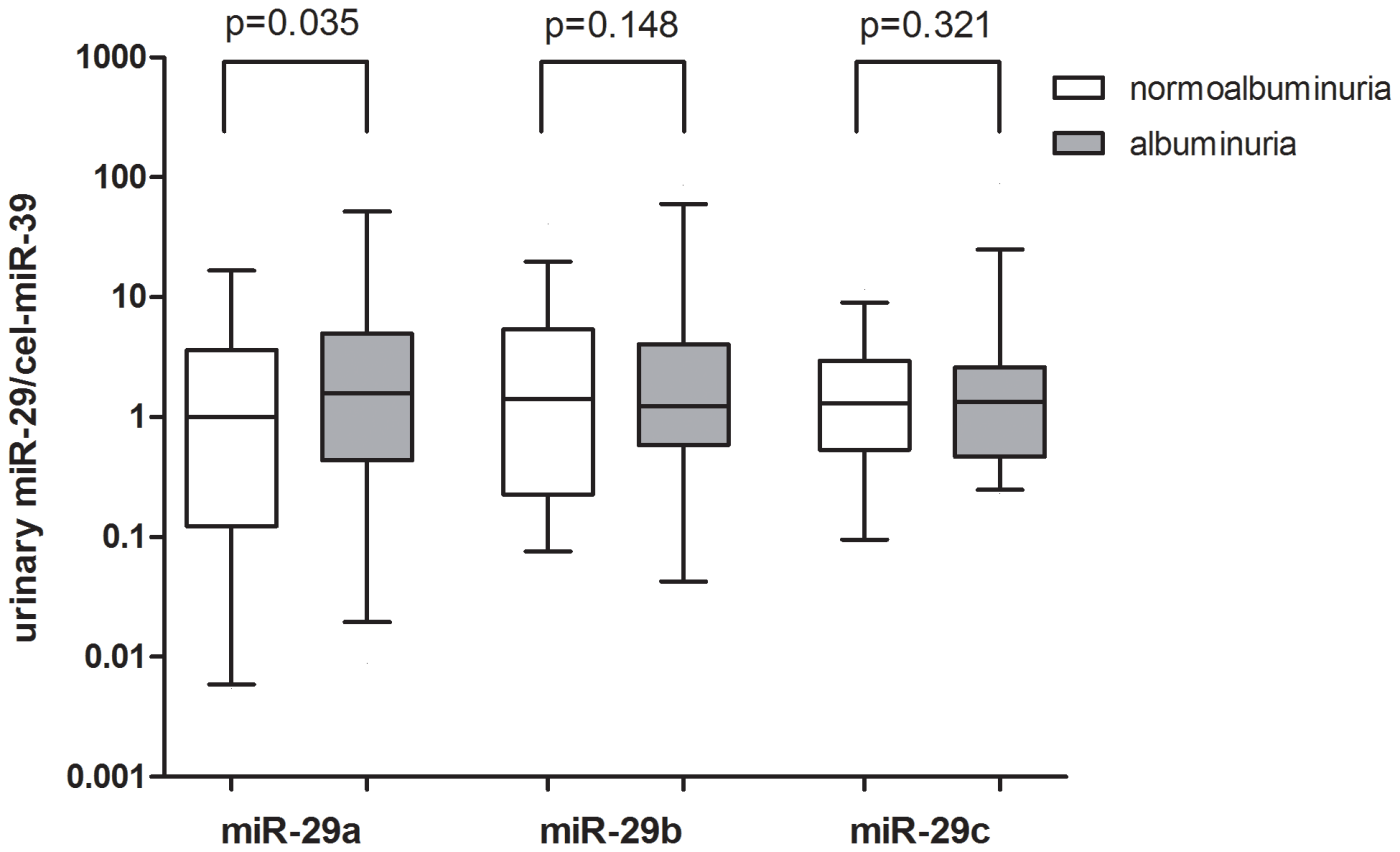
Comparison of urinary miR-29 members between diabetes patients with albuminuria and normoalbuminuria. Urinary miR-29a was higher in diabetes with albuminuria group than in diabetes with normoalbuminuria group (p = 0.035). There was no significant difference in urinary miR-29b (p = 0.148) or miR-29c (p = 0.321) levels between two groups. The values are represented as ratio to the median of diabetes with normoalbuminuria group. Data are compared by Mann-Whitney U test.

We compared the abundance of miR-29 family in urinary supernatant, as shown in Fig 1., miR-29a and miR-29c levels are significantly higher than miR-29b in urinary supernatant of type 2 diabetes (both with a p value <0.001). Urinary miR-29 levels in diabetes with normoalbuminuria and albuminuria groups are compared in Fig 2. Urinary concentration of miR-29a in diabetes with albuminuria group was higher (p = 0.035) than that of diabetes with normoalbuminuria group. No significant difference was found in urinary miR-29b (p = 0.148) or miR-29c (p = 0.321) levels between two groups.

### Correlation with Albuminuria and Renal Function

**Figure 3:**
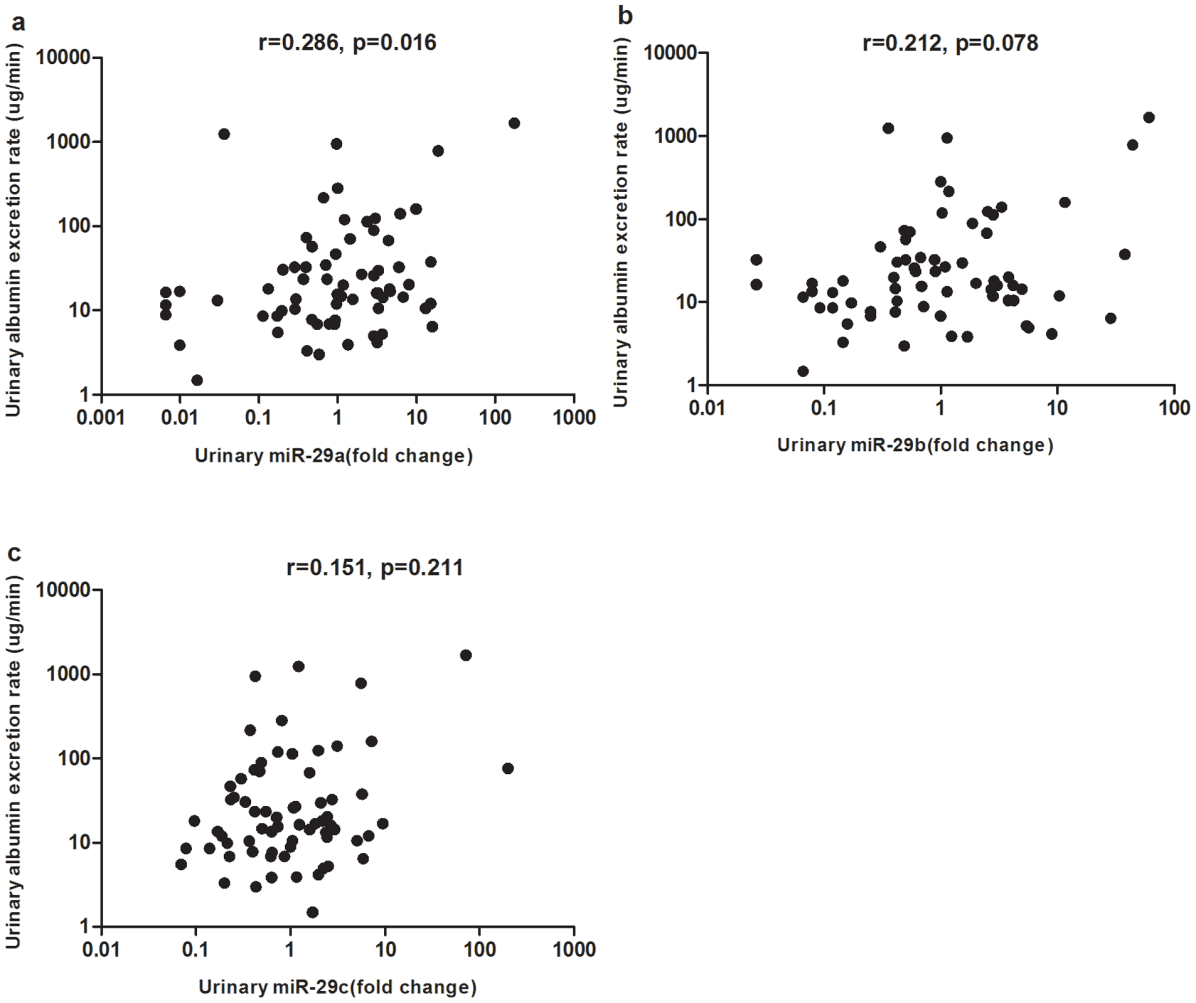
Correlation between urinary miR-29 members and urinary albumin excretion rate. a: Urinary miR-29a significantly correlated with urinary albumin excretion rate (r = 0.286, p = 0.016). b: Correlation between urinary albumin excretion rate and miR-29b was borderline significant (r = 0.212, p = 0.078). c: There was no significant correlation between miR-29c and urinary albumin excretion rate (r = 0.151, p = 0.211). Data were compared by Spearman’s rank order correlations.

The relation between urinary miR-29 levels and clinical parameters are further investigated. As shown in Fig. 3, we found that urinary albumin excretion rate significantly correlated with miR-29a (r = 0.286, p = 0.016), however, its correlation with miR-29b was borderline significant (r = 0.212, p = 0.078), while no significant correlation (r = 0.151, p = 0.211) was found between miR-29c and urinary albumin excretion rate. Correlations between miR-29 and other clinical parameters are listed in Table 2. However, there were no significant correlations between urinary miR-29 and renal function (indicated by urea, creatinine, cystatin, β2-microglobulin and eGFR).

**Table 2:** Correlation between miR-29 family and renal function parameters.

	miR-29a		miR-29b		miR-29c	
	r	p	r	p	r	p
Urea	−0.004	0.968	0.119	0.283	0.021	0.851
Creatinine	−0.119	0.285	0.032	0.774	−0.007	0.953
β2-microglobulin	−0.114	0.344	0.070	0.562	−0.085	0.479
Cystatin	−0.105	0.376	0.060	0.612	−0.076	0.521
eGFR	0.118	0.290	−0.045	0.688	−0.056	0.612

### Correlation with Carotid Intima-mediaThickness (cIMT)

**Figure 4:**
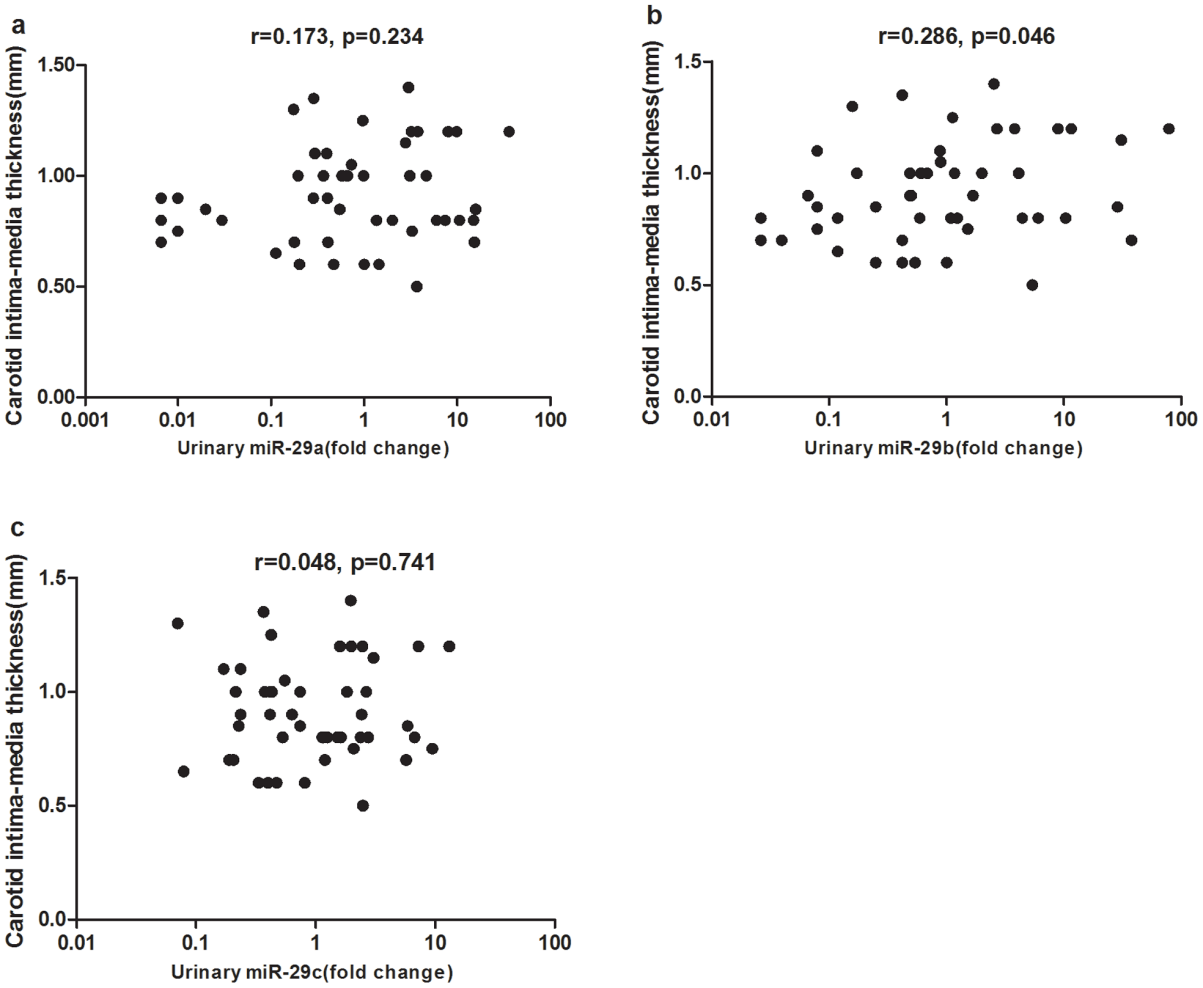
Correlation between urinary miR-29 members and carotid intima-media thickness (cIMT). a: There was no significant correlation between urinary miR-29a and carotid intima-media thickness (cIMT) (r = 0.173, p = 0.234). b: Urinary miR-29b significantly correlated with cIMT (r = 0.286, p = 0.046). c: There was no significant correlation between urinary miR-29c and cIMT (r = 0.048, p = 0.741) levels. Data were compared by Spearman’s rank order correlations.

MiR-29 is involved in the pathogenesis of insulin resistance, thus may play a crucial role in atherosclerosis. Here, we explore the relation between urinary miR-29 and carotid atherosclerosis. As revealed in Fig. 4, we found urinary miR-29b significantly correlated with carotid intima-media thickness (cIMT) (r = 0.286, p = 0.046). No significant difference were found between cIMT and miR-29a (r = 0.173, p = 0.234) or miR-29c (r = 0.048, p = 0.741) levels.

## Discussion

In this study, we found that the prevalence of hypertention is higher in type 2 diabetes patients with albuminuria than in those with normoalbuminuria. This is in accord with our knowledge that hypertention is highly prevalent in patients with diabetes and its development coincides with that of hyperglycaemia [22]. Hypertention is considered the risk factor of chronic kidney disease (CKD) in type 2 diabetes [23]. We also found that type 2 diabetes patients with albuminuria have a higher prevalence of diabetic retinopathy compared to those with normoalbuminuria. This is consistent with previous findings on concordance of diabetic retinopathy and diabetic nephropathy in type 1 and type 2 diabetes [24]–[26]. Diabetic retinopathy was considered useful in diagnosing and screening of diabetic nephropathy in type 2 diabetes [24], similarly, microalbuminuria was an independent risk factor for development and progression of diabetic retinopathy [25]. The common etiologic basis may explain the coexistence of both diabetic microvascular complications [26].

The relative higher abundance of miR-29a and miR-29c compared with miR-29b in urine supernatant is similar to the intracellular trend observed in proximal tubular cells (NRK52E) and mouse kidney tissue [13]. The differential stability and posttranscriptional processing of each mature miR-29 family member may explain the distinct abundance [27].

Previous studies of miR-29 on kidney disease revealed downregulated expression of miR-29a and miR-29c in kidneys of early and advanced STZ-diabetic nephropathy models [13] and decreased miR-29c level in kidneys of humans with IgA nephropathy and rats with renal interstitial fibrosis [13], [28]. Downregulation of miR-29 family correlated with increases in kidney fibrosis [13], [28]. Gang et al reported that miR-29b and miR-29c in urine sediment were decreased in patients IgA nephropathy, and urinary miR-29b and miR-29c correlated negatively with albuminuria and positively with renal function [29]. All the combined observations suggested that downregulation of intracellular miR-29 family mediated the pathogenesis of diabetic nephropathy and IgA nephropathy. However, miR-29c was reported to be up-regulated in kidney glomeruli from db/db mice and in vivo knock-down of miR-29c ameliorated the progression of diabetic nephropathy [12]. The use of two different animal models: STZ-induced type 1 diabetes in C57BL mice and db/db mice imitating type 2 diabetes may explain the converse results in two studies [12], [13]. This converse findings may also suggest that pathogenesis of diabetic nephropathy in type 2 diabetes may not be exactly the same with that of type 1 diabetes. In our report, we found that urinary miR-29a was elevated in subjects with albuminuria compared to those with normoalbuminuria, and positively correlated with albuminuria in type 2 diabetes subjects. This is different from what Lv et al reported recently. They found that when compared with controls, miR-29 family levels were decreased in exosome isolated from urine of CKD patients, including those with biopsy proven diabetic nephropathy and miR-29c correlated positively with eGFR and negatively with degree of tubulointerstitial fibrosis [30]. However, there is one drawback in their research, they used RNU6B as endogenous control for determination of miR-29 levels in urinary exosome but the authors provided no evidence that RNU6B was constantly expressed in each urine specimen and did not prove it to be suitable as normalization. Furthermore, Hanke et al reported that urinary RNU6B was higher in patients with bladder cancer and those with urinary tract infection compared with healthy subjects, they also found that RNU6B was not detectable in 8.5% of the urine samples, suggesting that RNU6B was not suitable for normalization of miRNAs levels in urine [31]. Since the urinary miRNA expression profiles vary in different disease condition, the ideal approach is to spike into the urine samples with synthetic, nonhuman mature miRNA at the beginning of RNA extraction as normalization [32]. As in our research, synthetic miRNA from C. elegans, cel-miR-39 was used as a spike-in control, the different controls used for normalization and different origin of urinary miRNAs (urine supernatant vs urinary exosome) may contribute to the converse alteration of urinary miR-29s observed in these two studies. Since circulating miRNAs mainly derive from cellular release, circulating miRNAs were generally considered to have the same trend of alteration, either increase or decrease with that in tissue of patients with various types of cancer even though converse trend of alteration was also observed [32]. An inverse correlation between urinary supernatant miRNA and intracellular miRNA has been reported by Gang et al, they found that miR-155 was decreased in urine sediment but increased in urine supernatant in patients with bladder cancer [33]. However, in our study, we did not detect the intrarenal expression of miR-29 family, therefore, it is not clear whether the trend of alteration of miR-29 in urinary supernatant is consistent with that in kidney.

Another intriguing finding is that urinary miR-29b positively correlated with cIMT in type 2 diabetes, but no significant correlation was found between urinary miR-29b and albuminuria or eGFR, this finding suggests the possibility that miR-29b might be implicated in the pathogenesis of atherosclerosis. Previous functional study on miR-29s focused on its great anti-fibrotic effects based on targeting a variety of collagens and important role of miR-29 in regulation of cell differentiation, proliferation and apoptosis [27]. The potential role of miR-29 in atherosclerosis has not been reported yet. Insulin resistance mediated by miR-29 deregulation might explain the correlation between urinary miR-29b and atherosclerosis. Insulin induces translocation of glucose transporter GLUT4 from intracellular vesicles to the plasma membrane through Akt activation subsequently potentiate the glucose transport [34]. Akt -1, -2, -3 are three isoforms of Akt in insulin sensitive tissue, and defective signaling through Akt-2 and -3 mediates insulin resistance in human skeletal muscle [35]. Wei et al reported that AKT3 was direct target of miR-29 with conserved miR-29 binding site in its 3’UTR and there was an inverse relationship between miR-29 and AKT3 levels skeletal muscle in vivo [36]. Furthermore, miR-29 was highly expressed in muscle, fat and liver of type 2 diabetes rats and overexpression of miR-29 attenuated insulin-induced Akt activation and glucose import [15]. Insulin resistance was an independent predictor of atherosclerosis in obese patients, thus upregulation of miR-29 may indirectly contribute to atherosclerosis [18]. In addition, endothelial nitric oxide synthase (eNOS) is one of Akt targets and insulin-induced activation of the PI3K-Akt-eNOS pathway in vascular endothelial cells was considered antiatherosclerotic through increased generation of NO, vasorelaxation, and suppressed expression of vascular cell adhesion molecule-1 [37]. Complete loss of insulin signal in vascular endothelial cells aggravated atherosclerosis in endothelium-specific insulin receptor–deficient apolipoprotein E null mice [38]. All the combined results together with our findings suggest that miR-29 deregulation may be implicated in pathogenesis of atherosclerosis via systemic and endothelial insulin resistance in type 2 diabetes. In this study, we firstly reported the possibility of urinary miR-29b as potential biomarker of atherosclerosis even though more evidence including in vitro and in vivo studies is needed to validate the role of miR-29 in atherosclerosis.

There are several limitations that should be acknowledged in this study. First, renal biopsy was not performed in each subject with type 2 diabetes, therefore, we did not get the correlation between urinary miR-29 and renal structure such as degree of renal fibrosis, thus unable to fully evaluate the feasibility of urinary miR-29 as biomarkers of diabetic nephropathy. Second, we did not detect miR-29 levels in serum, since miR-29 in urine supernatant may derive from glomerular ultrafiltration or renal excretion, the direct source of urinary miR-29 was not clear. However, this limitation does not refute the potential use of urinary miR-29 as surrogate biomarkers if they correlate with defined end points including atherosclerosis and diabetic nephropathy. Third, we did not fully elucidate the underlying mechanism of the correlation between urinary miR-29 and cIMT. The potential role of miR-29 in atherosclerosis needs to be further explored in basic research.

## Conclusions

Urinary miR-29a was increased in type 2 diabetes patients with albuminuria compared to those with normoalbuminuria and urinary miR-29a positively correlated with the degree of albuminuria. We also found a positive correlation between urinary miR-29b and carotid intima-media thickness (cIMT) in type 2 diabetes. Therefore, they may have the potential to serve as alternative biomarker for diabetic nephropathy and atherosclerosis in type 2 diabetes. The underlying mechanism by which urinary miR-29 correlated with diabetic vascular complications needs further exploration.
